# Spindle Cell Lipoma Occurring in the Buccal Mucosa: An Unusual Location of This Benign Lipomatous Neoplasm

**DOI:** 10.1155/2015/805730

**Published:** 2015-09-30

**Authors:** Noala Vicensoto Moreira Milhan, Ana Sueli Rodrigues Cavalcante, Yonara Maria Freire Soares Marques, Yasmin Rodarte Carvalho, Ana Lia Anbinder

**Affiliations:** Department of Bioscience and Oral Diagnosis, Institute of Science and Technology (ICT), Universidade Estadual Paulista (UNESP), Avenida Engenheiro Francisco José Longo 777, 12245-000 São José dos Campos, SP, Brazil

## Abstract

Spindle cell lipoma is a benign lipomatous neoplasm, which rarely occurs in the oral cavity. The aims of this paper are to report a case of spindle cell lipoma located in buccal mucosa and discuss the main clinical, histological, and immunohistochemical findings of this entity. Thus, we report a 4-year history of an asymptomatic smooth surface nodule in an elderly Caucasian man with clinical hypothesis of fibroma. The histopathological examination showed spindle cells, mature adipose tissue, and many mast cells in a stroma of connective tissue presenting ropey collagen fibers bundles. After immunohistochemical analysis, the final diagnosis was spindle cell lipoma.

## 1. Introduction

Spindle cell lipoma (SCL) is a histological variant of lipoma that often occurs in the neck, back, and shoulder [1]. It is rare in the oral cavity [2] and only 35 well-documented cases were published between 1984 and 2012 in the oral region [3]. Usually SCL is less than 5 cm in size, affects mainly elderly men [1, 3], and has a benign clinical course [1]. Generally, SCL is removed without imaging examination because it is usually found in superficial locations and is small in size [4].

In this paper, we report a case of a spindle cell lipoma located in the buccal mucosa and discuss important features of this rare oral lesion.

## 2. Case Report

A 64-year-old Caucasian man presented with a 4-year history of an asymptomatic nodular soft tissue mass located in the right buccal mucosa. Clinical examination of oral mucosa revealed a pedunculated and well-circumscribed nodule with about 1 cm in diameter. The nodule had fibrous consistency, smooth surface and it was covered by undamaged mucosa. The clinical hypothesis was traumatic fibroma.

**Figure 1:**
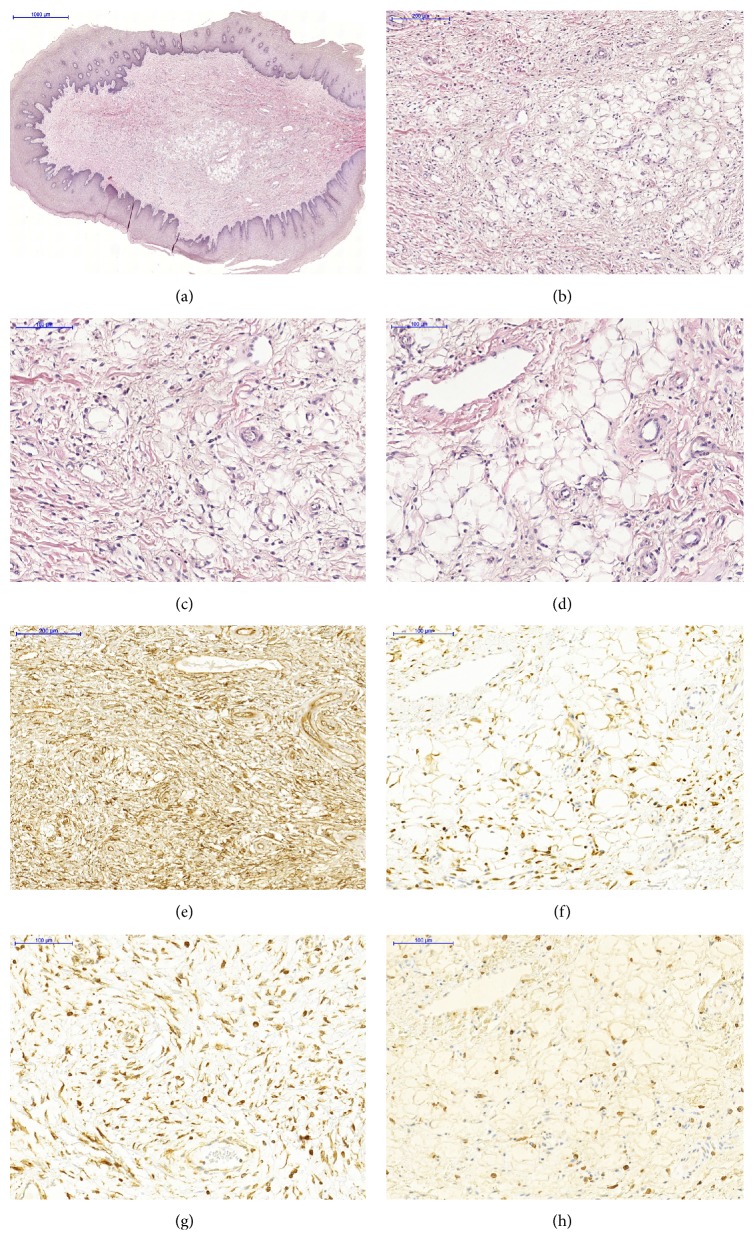
A circumscribed fibrous lesion covered by acanthotic epithelium showing lipomatous content at the centre of connective tissue (a). Mature adipose tissue in a background presenting ropey collagen fibers bundles (b). Spindle cells, vessels, and mast cells near lipomatous content ((c)-(d)). Spindle cells were strongly positive for CD-34 antibody (e) and positive for Bcl-2 (f) and vimentin antibodies (g). The great number of mast cells was demonstrated by mast-cell staining (h). ((a) to (d)) Hematoxylin and eosin stain; ((d) to (h)) immunohistochemistry stain.

Excisional biopsy of the nodule was performed and histopathological examination revealed connective tissue fragments presenting spindle cells in a background with ropey collagen fibers bundles. A diffuse infiltrate of mast cells was observed. In some slices, a central area of mature adipose tissue, with normal aspect, was noted. The lesion was well vascularized and covered by stratified squamous epithelium with areas of acanthosis, hyperplasia, duplication of basal cell layer, and hydropic degeneration (Figures 1(a)–1(d)).

After initial microscopic examination, the diagnoses hypothesis included traumatic fibroma, neurofibroma, and SCL. Immunostaining of the specimen with primary antibodies against vimentin (VIM, Clone V9-Dako), CD-34 (Clone QBEnd10-Dako), S-100 (Clone S100-Dako), Bcl-2 (Clone 124-Dako), smooth muscle actin (SMA, Clone 1A4-Dako), and mast cell (Clone AA1-Imgenex) was performed. The immunohistochemical reactions revealed negativity of spindle cells for S-100 and SMA and positivity for CD-34 (Figure 1(e)), Bcl-2 (Figure 1(f)), and VIM (Figure 1(g)). Moreover, mast-cell immunohistochemistry staining was performed and confirmed the presence of these cells (Figure 1(h)). These findings were consistent with the diagnosis of SCL. The patient has been followed up and no recurrence occurred after one year.

## 3. Discussion

The World Health Organization (WHO) categorizes benign lipomatous entities into some distinct lesions: lipoma, lipoblastoma, lipomatosis, lipomatosis of a nerve, myolipoma of soft tissue, angiolipoma, spindle cell/pleomorphic lipoma, chondroid lipoma, and hibernoma [5].

SCL was first described by Enzinger and Harvey in 1975. This lesion is usually located in the subcutaneous tissues of the neck, back, and shoulder [1]. In contrast, SCL is rare in the oral region [3] and comprises only about 1,62% of intraoral lipomatous tumors [2]. SCL has been reported in the tongue, buccal mucosa, floor of the mouth, hard palate, lip, and alveolar ridge [3]. One case in the maxilla was also reported [6].

SCL has a male predilection (male : female, 10 : 1) and the mean age of occurrence is 56 years [1]. In oral cavity cases, the male predilection is 2 : 1, while the mean age of occurrence is about 55 [3]. Usually, SCL appears as a well-circumscribed and painless mass [1] that generally has less than 5 cm in diameter [1, 3], although it may reach more than 10 cm [1, 4].

When SCL occurs on the face it may be misdiagnosed as an atypical lipomatous tumor because in this area the infiltrative behavior of SCL is common, such as malignant tumors. This infiltration occurs probably due to facial anatomical features, which involve poorly demarcated fascial planes [7]. SCL is generally solitary, although patients with bilateral SCLs of the tongue [8, 9] and nose [7] and lesions located simultaneously in upper region of the back and axilla [10] have been reported. Multiple SCLs may be familial or nonfamilial [11]. The treatment for SCL is local excision, usually without local recurrence [1, 3].

Histologically, SCL is composed of spindle/fusiform cells that may present a parallel arrangement, showing nuclear palisading. The stromal background shows ropey collagen fibers bundles. This lesion may also have myxoid areas. In association with spindle cells, different amounts of adipose tissue are usually observed. Moreover, the presence of mast cells in SCL is common [1, 5]. The vascular pattern usually is inconspicuous but there are lesions with prominent vascular pattern [1].

SCL is also called pleomorphic lipoma because it may show pleomorphic or bizarre multinucleated giant cells, sometimes with floret-like arrangement of the nuclei. Moreover, fat cells with varying size may be observed in SCL [5].

Some SCLs show several histological differences from classic SCL and have been proposed as variants. SCL presenting prominent fibrous tissue was described as fibrous SCL. This feature may represent one end of the histopathological spectrum of SCL, once the spindle cells are able to produce significant amounts of collagen [12]. SCL showing irregular and sometimes cleft-like spaces and villiform projections was classified as angiomatous. The cells of these irregular spaces may present vascular-lymphatic differentiation; however lymphatic phenotype is more common than vascular one [13].

Spindle to stellate cells, with cytoplasmic dendritic processes, and adipose tissue in a myxoid background have been observed in a recently described lesion known as dendritic fibromyxolipoma. However, some studies have proposed that this lesion is just a variant of the SCL [14, 15]. Wong et al. [15] performed cytogenetic analysis and concluded that this lesion does not represent a separate entity.

In a classic SCL, the ratio of mature adipocytes and spindle cells is variable; however the majority of cases have high amounts of both components. There are low-fat and fat-free SCLs, but they are less common than classic SCLs. In a previous study that evaluated over 300 cases of SCLs, only 34 low-fat and fat-free SCLs were identified. Moreover, only 3 cases of low-fat and fat-free SCLs were considered as a diagnosis, showing that different proportions of histopathologic elements, especially adipose tissue, hamper the diagnosis [16].

The histological differential diagnosis of SCL depends on features that predominate in each lesion. It usually includes benign tumors such as classic lipoma, schwannoma, neurofibroma [7], leiomyoma [1], and solitary fibrous tumor [16]. However, atypical lipomatous tumors (ALT) may also represent a differential diagnosis of SCL [7]. Dermatofibrosarcoma protuberans and subconjunctival herniated orbital fat are malignant [17] and benign [18] lesions, respectively, which may be confused with SCL. However, subconjunctival herniated orbital is a specific eye condition and only 2 cases of intraoral dermatofibrosarcoma protuberans have been described in literature [17]. Then, these lesions usually are differential diagnosis of extraoral SCLs.

Less commonly, ALT, also known as well-differentiated liposarcoma, may also be a differential diagnosis of SCL [7, 19]. SCL may be confused with ALT; however there are histological components that may help to differentiate ALT from benign lesions: ALT presents fibrous septa with atypical stromal cells demonstrating nuclear hyperchromasia, significant variation in adipocyte shape and size, which includes enlarged and atypical adipocyte nuclei. Moreover, ropey collagen fibers have not been observed in ALT [7, 20]. In cases where the histological evaluation is not sufficient to differentiate SCL from ALT, the positive immunoreactivity for MDM2, CDK4, p16, and Rb in ALT and not in SCL may be helpful in the final diagnoses [21].

In this study, after initial microscopic evaluation the hypotheses were benign tumors, which included traumatic fibroma, neurofibroma, and SCL. SCL was the last one to be considered because the central area of adipose tissue was present only in some slices. Considering the proliferation of spindle cells in a collagenized background, traumatic fibroma was a hypothesis. The spindle cell component and the numerous mast cells contributed to the hypothesis of neurofibroma. When the slices showing adipose tissue were observed, SCL was also considered. However, we could not eliminate the hypotheses of traumatic fibroma and neurofibroma without immunohistochemical analysis. A previous study that evaluated 320 cutaneous neurofibromas showed that 6.9% presented intratumoral mature fat [22].

The immunohistochemical analysis was accomplished and the positivity of spindle cells for CD-34 excluded the possibility of a traumatic fibroma [23] while their negativity for S-100 excluded the possibility of a neurofibroma [22]. Furthermore, the spindle cells showed positivity for VIM and Bcl-2 and negativity for SMA which was compatible with the immunohistochemical panel of SCL [24]. The SMA negative staining excluded the muscular and myofibroblastic origin of spindle cells [25]. Thus, considering the histological and immunohistochemical findings, our diagnosis was classic SCL.

In spite of the fact that adipose tissue has been seen only in some areas, it was more than 5% of the lesion. Low-fat and fat-free SCL are considered to be tumors in which mature adipocytes are less than 5% of the tumor [16].

In summary, SCL is a rare lesion in the oral cavity that may be confused with other entities. In this report we presented a rare case of a classic SCL to make clinicians and pathologists aware of this lesion in the oral cavity and discussed the possible differential diagnoses.
